# Supplementary material for: A Polymorph of Dipeptide Halide Glycyl-L-Alanine Hydroiodide Monohydrate: Crystal Structure, Optical Second Harmonic Generation, Piezoelectricity and Pyroelectricity
Source: Materials (Basel). 2023 May 12;16(10):3690. doi: 10.3390/ma16103690 (PMC10221006; doi:10.3390/ma16103690)
Supplement: Supplementary file 1 [file materials-16-03690-s001.zip › materials-2306005-supplementary.pdf]

A polymorph of dipeptide halide Glycyl-L-Alanine Hydroiodide Monohydrate: crystal structure, optical second harmonic generation, piezoelectricity and pyroelectricity

Rosa M. F. Baptista [1], Clara S. B. Gomes [2,3,4], Bruna Silva [1], João Oliveira [1], Bernardo Almeida [1], Cidália Castro [5], Pedro V. Rodrigues [5], Ana Machado [5], Ruben B. Freitas [6], Manuel R. L. F. Rodrigues [1], Etelvina de Matos Gomes [1] and Michael Belsley [1,*]

[1] Centre of Physics of Minho and Porto Universities (CF-UM-UP), Laboratory for materials and Emergent Technologies (LAPMET), University of Minho, Campus de Gualtar, 4710-057 Braga, Portugal
[2] LAQV-REQUIMTE, Department of Chemistry, NOVA School of Science and Technology, NOVA University Lisbon, 2829-516 Caparica, Portugal
[3] UCIBIO, Department of Chemistry, NOVA School of Science and Technology, NOVA University Lisbon, 2829-516 Caparica, Portugal
[4] i4HB, NOVA School of Science and Technology, NOVA University Lisbon, 2829-516 Caparica, Portugal
[5] Institute for Polymers and Composites, University of Minho, Campus de Azurém, 4800-058 Guimarães, Portugal
[6] Departament of Electronic Engineering, Universidade do Minho, Campus de Gualtar, 4710-057 Braga, Portugal
* Correspondence: belsley@fisica.uminho.pt

Supplementary Information

S1. Crystal Structure

Table S1. Bond lengths [Å] and angles [°] for Gly-L-Ala.HI.H_2O (Poly2).

Bond lengths		*Bond angles*	
C(1)-N(1)	1.481(4)	N(1)-C(1)-C(2)	109.9(4)
C(1)-C(2)	1.532(6)	N(1)-C(1)-H(1A)	109.7
C(1)-H(1A)	0.9700	C(2)-C(1)-H(1A)	109.7
C(1)-H(1B)	0.9700	N(1)-C(1)-H(1B)	109.7
C(2)-O(1)	1.243(6)	C(2)-C(1)-H(1B)	109.7
C(2)-N(2)	1.323(7)	H(1A)-C(1)-H(1B)	108.2
C(3)-N(2)	1.459(7)	O(1)-C(2)-N(2)	124.5(5)
C(3)-C(5)	1.521(8)	O(1)-C(2)-C(1)	120.9(4)
C(3)-C(4)	1.537(9)	N(2)-C(2)-C(1)	114.6(4)
C(3)-H(3)	0.9800	N(2)-C(3)-C(5)	109.7(5)
C(4)-H(4A)	0.9600	N(2)-C(3)-C(4)	111.7(5)
C(4)-H(4B)	0.9600	C(5)-C(3)-C(4)	110.4(5)
C(4)-H(4C)	0.9600	N(2)-C(3)-H(3)	108.3
C(5)-O(2)	1.221(7)	C(5)-C(3)-H(3)	108.3
C(5)-O(3)	1.316(7)	C(4)-C(3)-H(3)	108.3

N(1)-H(10A)	0.88(3)	C(3)-C(4)-H(4A)	109.5
N(1)-H(10B)	0.85(3)	C(3)-C(4)-H(4B)	109.5
N(1)-H(10C)	0.88(3)	H(4A)-C(4)-H(4B)	109.5
N(2)-H(20)	0.89(10)	C(3)-C(4)-H(4C)	109.5
O(3)-H(30)	0.91(10)	H(4A)-C(4)-H(4C)	109.5
O(4)-H(40)	0.8159(5)	H(4B)-C(4)-H(4C)	109.5
O(4)-H(41)	0.8283(5)	O(2)-C(5)-O(3)	123.9(5)
		O(2)-C(5)-C(3)	124.1(5)
		O(3)-C(5)-C(3)	111.9(5)
		C(1)-N(1)-H(10A)	106(4)
		C(1)-N(1)-H(10B)	109(4)
		H(10A)-N(1)-H(10B)	100(7)
		C(1)-N(1)-H(10C)	124(9)
		H(10A)-N(1)-H(10C)	106(8)
		H(10B)-N(1)-H(10C)	110(10)
		C(2)-N(2)-C(3)	124.4(4)
		C(2)-N(2)-H(20)	118(6)
		C(3)-N(2)-H(20)	117(6)
		C(5)-O(3)-H(30)	106(6)
		H(40)-O(4)-H(41)	99.17(2)

Table S2. Torsion angles [°] for Gly-L-Ala.HI.H_2O (Poly2), Gly-L-Ala.HI.H_2O (Poly1) and Gly-L-Ala.

	Poly 2	*Poly 1* [1]	*Gly-L-Ala* [2]
N(1)-C(1)-C(2)-O(1)	-14.2(6)		
N(1)-C(1)-C(2)-N(2)	167.9(5)	168.13	-163.54
N(2)-C(3)-C(5)-O(2)	-12.7(7)	-12.94	-35.88
C(4)-C(3)-C(5)-O(2)	110.8(7)		
N(2)-C(3)-C(5)-O(3)	167.9(5)	167.78	149.99
C(4)-C(3)-C(5)-O(3)	-68.5(6)		
O(1)-C(2)-N(2)-C(3)	-0.2(9)		
C(1)-C(2)-N(2)-C(3)	177.7(5)	177.88	-173.94
C(5)-C(3)-N(2)-C(2)	-126.2(5)	-126.27	-78.36
C(4)-C(3)-N(2)-C(2)	111.0(6)		

Table S3: Crystal data and structure refinement for Gly-L-Ala.HI.H_2O (Poly2).

	**Gly-L-Ala.HI.H_2O (Poly 2)**
Formula	$C_5H_{13}IN_2O_4$
M	292.07
λ (Å)	0.71073
T (K)	293(2)
crystal system	Monoclinic
space group	$P2_1$
Crystal description	Prism
Crystal color	Yellow
a (Å)	7.747(6)
b (Å)	6.435(5)
c (Å)	10.941(9)
α (deg)	90
β (deg)	107.53(3)
γ (deg)	90
V (Å^3)	520.1(7)
Z	2
ρ_{calc} (g cm^{-3})	1.865
μ (mm^{-1})	3.063
θ_{max} (deg)	28.284
total data	12554
unique data	2562
R_{int}	0.0551
R [I>3σ(I)]	0.0286
wR_2	0.0683
Goodness of fit	1.097
ρ_{min}	-1.752
ρ_{max}	0.695

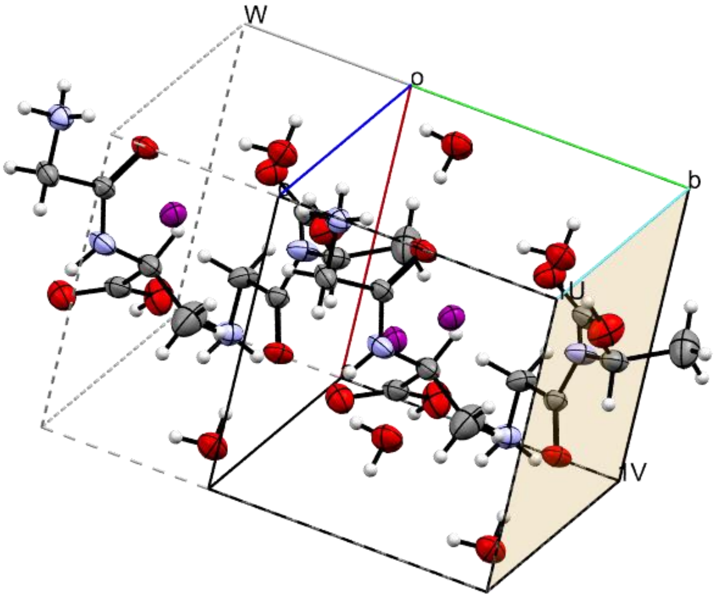

Figure S1. Unit cell of Gly-L-Ala.HI.H_2O (Poly2) showing the (010) plane, in yellow, perpendicular to the *b* axis.

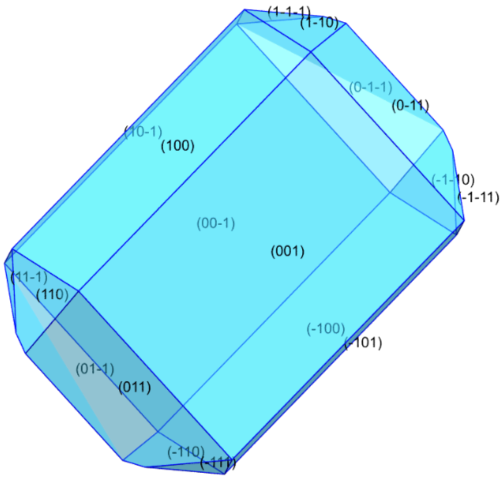

Figure S2. Gly-L-Ala.HI.H_2O (Poly2) morphology with (*hkl*) labels.

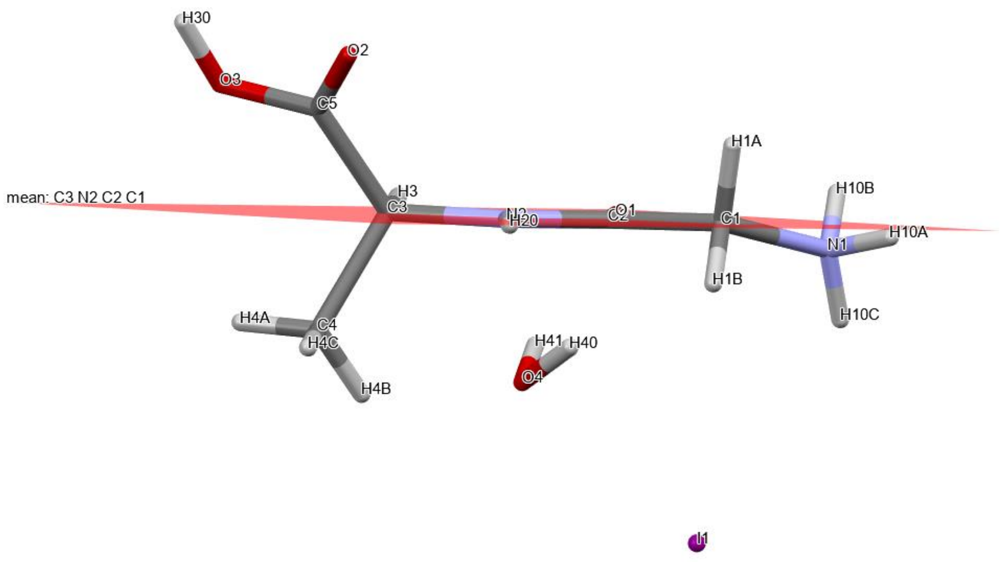

Figure S3. The main chain of the dipeptide molecule presents an almost planar *trans* conformation between atoms N1 and C3.

S2. Thermogravimetric analysis (TGA)

The TGA spectrum shows a small initial mass loss of 2% happening at 388 K that corresponding to the starting of water molecules evaporation. Above 473 K there is a considerable loss of mass (about 50%), correspondent to the degradation of the crystalline compound. The peak at 533 K corresponds to the fusion temperature of the dipeptide glycyl-l-alanine, is very close to the melting temperature reported for cyclo-glycyl-L-alanine which was 531 K. Therefore, the peak at 648 K, corresponds to the complete degradation of crystalline compound Gly-L-AlaHI.H_2O (Poly2).

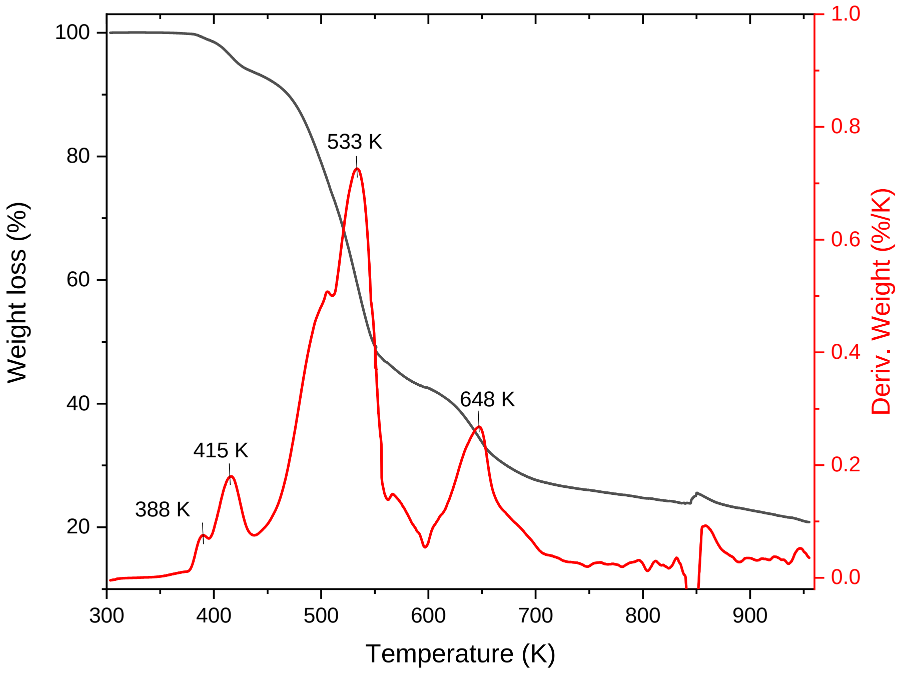

Figure S4. TGA spectra of synthesized glycyl-l-alanine iodide hydrate crystals.

S3. DSC analysis

In figure S5, the measured DSC of synthesized glycyl-l-alanine iodide hydrate crystals, shows one first peak at 363 K, area 5.55 J/g that indicates the starting of water loss in the structure. A second double peak shows at 388 K is followed by the maximum at 393 K with area 12.3 J/g corresponding to full loss of water. The cooling curve does not reproduce the heating curve, indicating that above 393 K the compound is not any more the initial one, according to what has just been stated before.

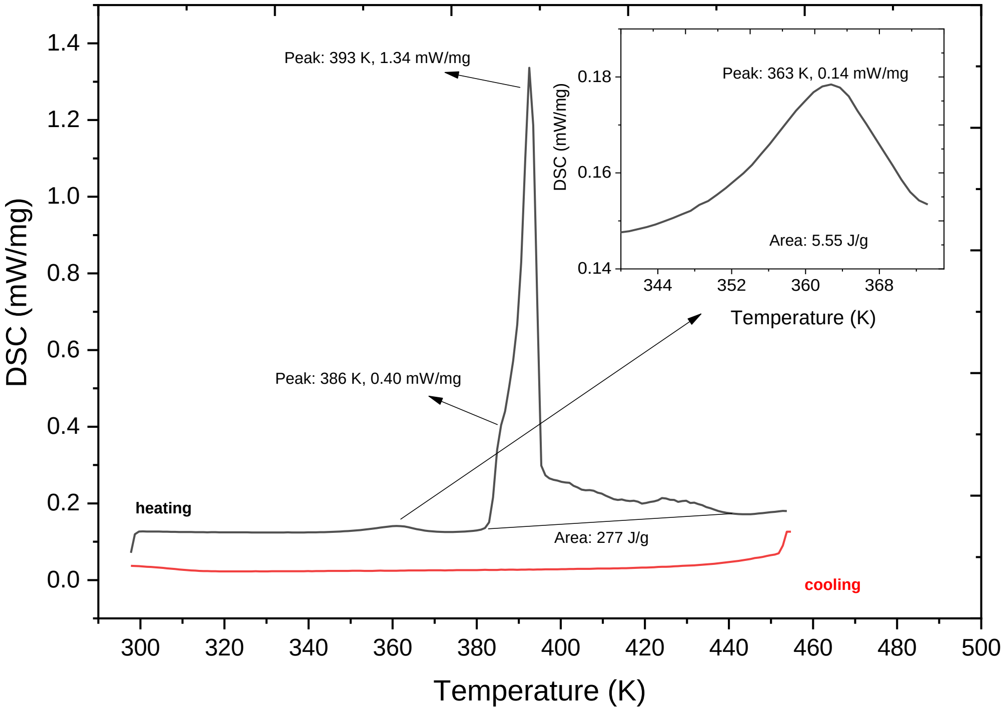

Figure S5. DSC spectra of synthesized glycyl-l-alanine iodide hydrate crystals.

S4. FTIR-ATR analysis

In Figure S6 the measured FTIR-ATR between 550-3800 cm^{-1} is shown for a glycyl-l-alanine iodide hydrate powder sample.

The intense and sharp band at 3475 cm^{-1} is assigned to the strong hydrogen-bonding established between internal water molecules and the oxygen atoms from the dipeptide bond. The N–H stretching bands are relatively weak and appear at 3232 cm^{-1} and 3188 cm^{-1}, while the C–H stretching bands are observed from 2960 to 3099 cm^{-1} region. Carbonyl stretching modes appear as sharp singlets at 1716 cm^{-1} and 1649 cm^{-1}. These results are in accordance to those reported in the literature for the glycyl-l-alanine molecule [3]. Deviation is observed in some bands, namely those related to asymmetric stretches in the terminal amine group, which can be explained by the fact that the dipeptide is in a different chemical environment due to its binding to the iodide ion and water molecule.

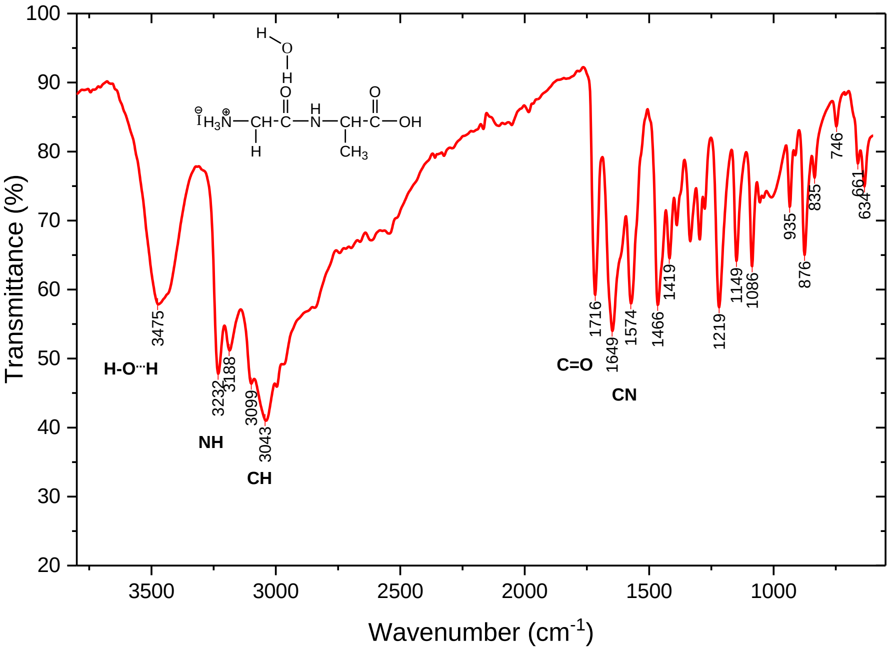

Figure S6. FTIR-ATR spectra of glycyl-l-alanine iodide hydrate powder between 550 – 3800 cm^{-1}.

S5. RAMAN Spectra

In Figure S7, the measured Raman modes between 50 – 800 cm^{-1}, 800 – 1800 cm^{-1} and 2700 – 3500 cm^{-1} are shown for glycyl-l-alanine iodide hydrate polycrystalline sample. The measurements were made under no polarized light (black), vertical (VV, red) and horizontal (VH, blue) polarized light. In the spectra, the low frequency modes under 500 cm^{-1} are due to crystal lattice vibrations. In the region 800 – 1800 cm^{-1} there are 3 peaks, for which the relative intensities are for VV polarization smaller (935 cm^{-1} and 1276 cm^{-1}) or higher for VH polarization (1090 cm^{-1}). These indicates that although the sample was polycrystalline there was some preferential orientation on the crystallite's distribution. It was not possible to compare the vibrations of the Raman modes with those of glycyl-l-alanine molecule because there is no information available in the literature.

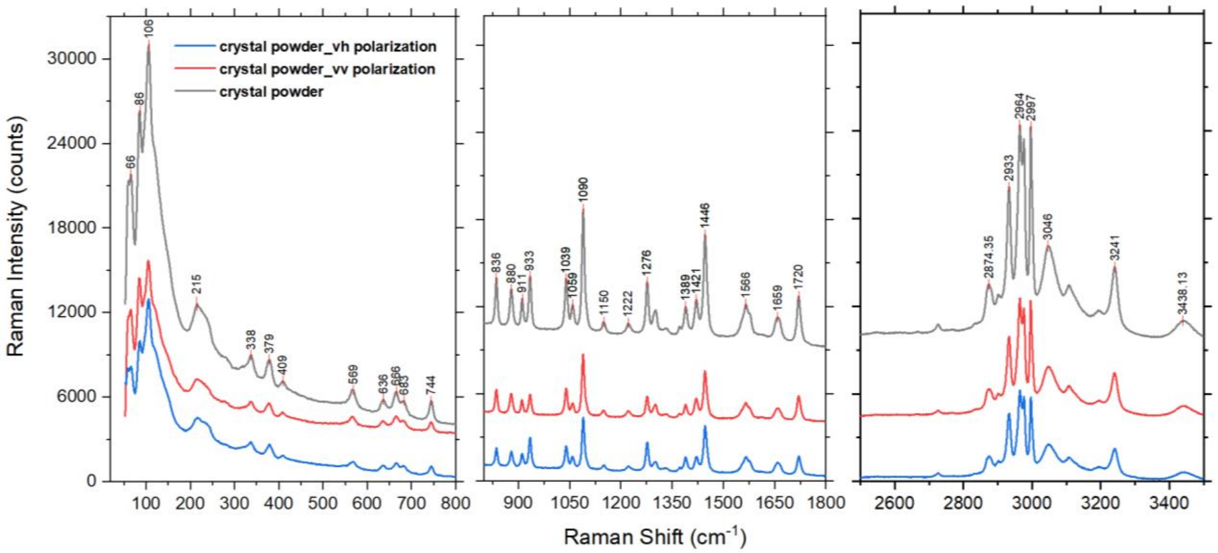

Figure S7. Raman spectra for glycyl-l-alanine iodide hydrate powder, expansions between 50 – 800 cm^{-1}, 800 – 1800 cm^{-1} and 2700 – 3500 cm^{-1}.

S6. Second Harmonic Measurements

Figure S8 shows a representative fit to the acquired second harmonic spectra that we expect to be approximately Gaussian as a function of frequency, i.e.

$$Counts = B + A\exp\left[-4\ln 2\left(\nu-\nu_0\right)^2/\Delta\nu^2\right]$$

For these data the fit yields a background B = -3.4 ± 0.3 Counts, an amplitude A = 828 ± 2 Counts, a central frequency ν_0 = 753.31±0.01 THz and a full width half maximum frequency of$\Delta\nu$ = 8.11 ± 0.02 THz. For reference, 759 THz corresponds to a wavelength of approximately 400nm:

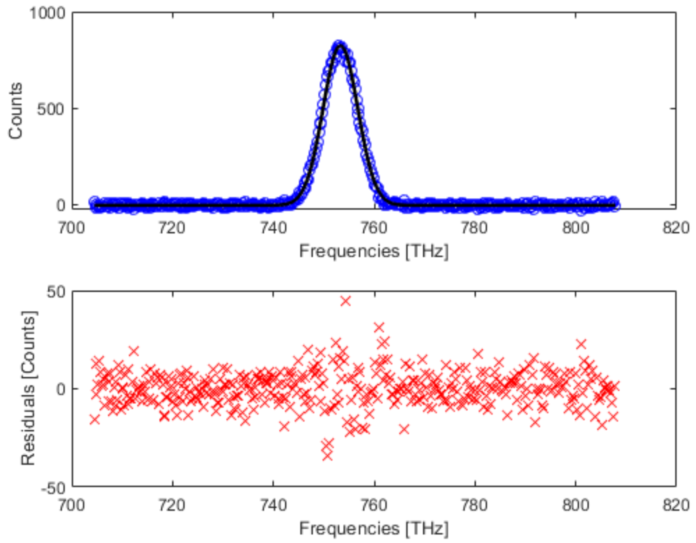

Figure S8. An example of the Gaussian fit to the obtained spectra. Blue circles represent the data points while the back line is the best least squares fit to the data.

For crystals belonging to point group 2, the nonlinear ssuceptibility tensor takes the form:

$$\begin{bmatrix} P_1^{2\omega} \\ P_2^{2\omega} \\ P_3^{2\omega} \end{bmatrix} = \begin{bmatrix} 0 & 0 & 0 & d_{14} & 0 & d_{16} \\ d_{21} & d_{22} & d_{23} & 0 & d_{25} & 0 \\ 0 & 0 & 0 & d_{34} & 0 & d_{36} \end{bmatrix} \begin{bmatrix} E_1^{\omega}E_1^{\omega} \\ E_2^{\omega}E_2^{\omega} \\ E_3^{\omega}E_3^{\omega} \\ 2E_2^{\omega}E_3^{\omega} \\ 2E_1^{\omega}E_3^{\omega} \\ 2E_1^{\omega}E_2^{\omega} \end{bmatrix} \qquad \text{(S1)}$$

Here, 1, 2 and 3 (or x , y and z respectively) are the electric field components of the incident laser beam in the frame of the crystal's principal dielectric axes with the crystallographic b axis taken to coincide with the direction of the dielectric y axis. If one assumes Kleinman sysmetry then $d_{21} = d_{16}$; $d_{25}=d_{36}=d_{14}$ and $d_{34}=d_{23}$.

For these biaxial crystals the two eigen polarizations for the slow ($\hat{e}_s$) and fast ($\hat{e}_f$) waves of a beam propagating at an aribitary direction described by the polar angles (θ,ϕ) relative to the crystal's dielectric z-axis are respectively:

$$\hat{e}_s = \begin{bmatrix} \cos\theta\cos\phi\cos\delta - \sin\phi\sin\delta \\ \cos\theta\sin\phi\cos\delta + \cos\phi\sin\delta \\ -\sin\theta\cos\delta \end{bmatrix} \qquad \text{(S2a)}$$

and

$$\hat{e}_f = \begin{bmatrix} -\cos\theta\cos\phi\sin\delta - \sin\phi\cos\delta \\ -\cos\theta\sin\phi\sin\delta + \cos\phi\cos\delta \\ -\sin\theta\sin\delta \end{bmatrix} \qquad \text{(S2b)}$$

Here δ is the angle between the polarization direction of the slow wave and the plane of propagation containing the z-axis [4]. Using these expressions it is possible to demonstrate that a fundamental beam propagating as an ordinary wave (extra-ordinary wave) incident along the dielectric y $(\theta = \phi = \pi/2)$ axis will not generate any extraordinary (ordinary) second harmonic light [4]. This precludes any chance of phase matching for beams travelling along the y dielectric axis due the dispersion between the fundamental and second harmonic waves. For beams that do propagate along or close to the y axis, appreciable second harmonic generation will in general only be observed when the incident beam waist is close to the exit surface.

To carry out the fit shown in Figure 15 of the main text, we followed the "double integral method" developed by Wang and Weiner [5] to describe second harmonic generation by ultrashort pulses in the simultaneous presence of spatial and temporal walk-off as well as phase mismatch. Assuming Gaussian temporal and spatial profiles with a transverse circular symmetry, Wang and Weiner arrive at the following expression for the energy of the second harmonic wave generated by a crystal of length L:

$$U_{2\omega} = \sqrt{\frac{\ln 2}{2\pi}} \frac{(2\omega)^2 d_{eff}^2}{\varepsilon_0 n_\omega n_{2\omega} \lambda_\omega c^3} \frac{U_\omega^2}{t_p z_R^2}$$
$$\int_o^L dz_1 \int_o^L dz_2 \frac{\exp\left[i\Delta k (z_1 - z_2)\right] \exp\left[-(z_1 - z_2)^2 / l_{S-T}^2\right]}{\left[1 - i(z_1 - z_0)/z_R\right]\left[1 + i(z_2 - z_0)/z_R\right]}$$

Here the energies of the fundamental and second harmonic pulses are U_ω and $U_{2\omega}$ respectively, t_p is the full width half maximum temporal pulse width, z_R and z_0 are the fundamental beam's Rayleigh distance and beam waist position in the crystal, λ_ω is the vaccum fundamental wavelength, while n_ω and $n_{2\omega}$ are the refracitve indices of the fundamental and second harmonic beams respectively. The parameter l_{S-T} represents the combined effects of spatial and temporal walk-off and is given by,

$$l_{S-T} = \left[\frac{\pi n_\omega \rho^2}{\lambda_\omega z_R} + \frac{\left(\alpha^2 + 16\right)\beta^2 \ln 2}{8t_p^2} \right]^{-1/2}$$

with ρ the spatial walk-off angle in radians, α characterizes the extent to which a linear chirp has broadened the pulse beyond the Fourier transform limit (t_0), $t_p^2 = \left(1+\alpha^2\right)t_0^2$, and β is the inverse group velocity mismatch between the fundamental and second harmonic beams. A nonlinear least squares minimization routine [6] was used to estimate the values of $z_0, z_R, \Delta k$ and l_{S-T}. The results corresponding to the fit shown in figure 15 are, $z_0 \approx 0.89\mu m$, $z_R \approx 16.4\mu m$, $\Delta n = \Delta k \lambda_\omega / 2\pi \approx 0.16$ with l_{S-T} being so large (above 500 μm) that it has a negligible effect.

We used a 2 mm Barium Beta Borate (BBO) to calibrate our second harmonic microscope. BBO has a walk-off angle of 68.4 mrad, an inverse group velocity mismatch of $\beta = 197\, fs/mm$ and we estimate that the linear chirp after passing through the microscope objective corresponds to a value of $\alpha \approx 0.8$. Assuming the same value for the Rayleigh distance as given by the above fit for the glycyl-L-alanine dipeptide crystal, numerical integration of the double integral for BBO gives a peak value of 1560. In contrast, the peak value for the glycyl-L-alanine dipeptide crystal using the above estimated values is only 0.53 due to the large phase mismatch.

S7. SEM and XRD of fibers

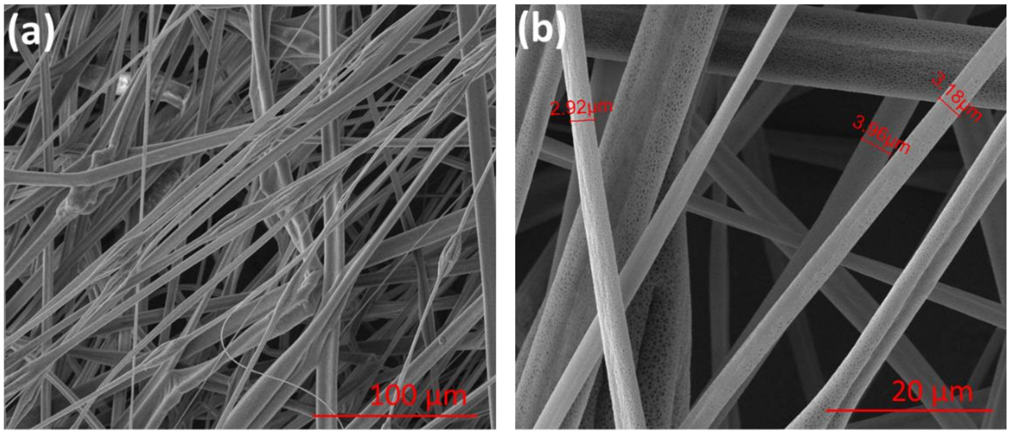

Figure S9. SEM images at magnification levels of 1000× (a) and 5000× (b) for PMMA with embedded Gly-L-AlaHI.H_2O (Poly2) crystals.

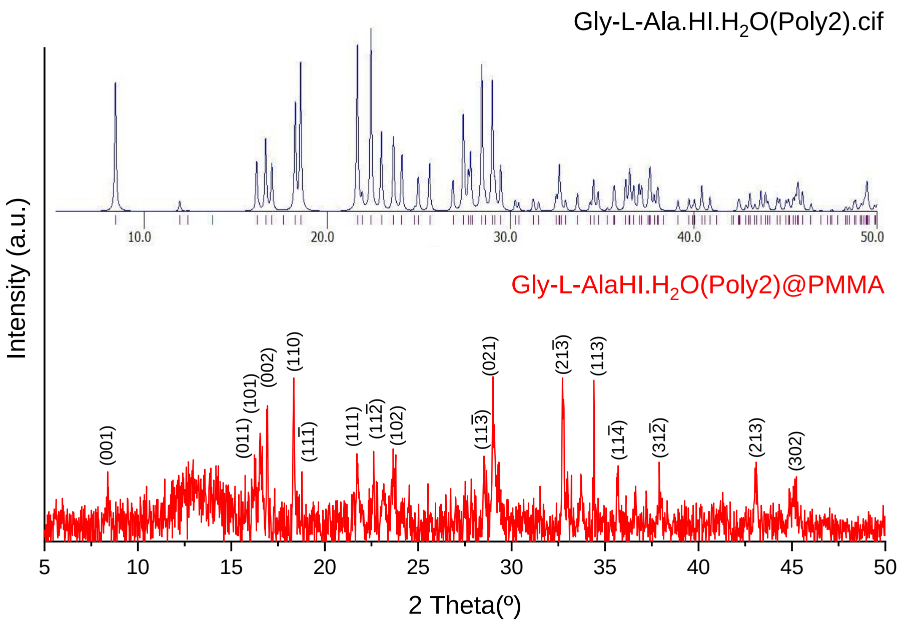

Figure S10. Measured X-ray diffraction pattern from a fiber array of Gly-L-Ala.HI.H_2O(Poly2)@PMMA. The inset shows the calculated powder pattern for crystalline Gly-L-AlaHI.H_2O (Poly2), from the crystallographic information file.

References

1. Kehrer, A.; Dou, S.-q.; Weiss, A. 79,81Br−127I−NQR, and Crystal Structure of Glycyl-l-alanine Hydrobromide Monohydrate and Hydroiodide Monohydrate. *Z. Naturforsch. A* **1989**, *44*, 659-668, doi:10.1515/zna-1989-0710.
2. Capelli, S.C.; Burgi, H.-B.; Dittrich, B.; Grabowsky, S.; Jayatilaka, D. Hirshfeld atom refinement. *IUCrJ* **2014**, *1*, 361-379, doi:10.1107/S2052252514014845.
3. Tulip, P.R.; Bates, S.P. First principles determination of structural, electronic and lattice dynamical properties of a model dipeptide molecular crystal. *Mol. Phys.* **2009**, *107*, 2201-2212, doi:10.1080/00268970903224955.
4. Tzankov, P.; Petrov, V. Effective second-order nonlinearity in acentric optical crystals with low symmetry. *Appl. Optics* **2005**, *44*, 6971-6985, doi:10.1364/ao.44.006971.
5. Wang, H.; Weiner, A.M. Efficiency of short-pulse type-I second-harmonic generation with simultaneous spatial walk-off, temporal walk-off, and pump depletion. *Ieee J. Quantum Elect.* **2003**, *39*, 1600-1618, doi:10.1109/JQE.2003.819531.
6. D'Errico, J. Fminspleas Available at MATLAB Central File Exchange. Available online: https://www.mathworks.com/matlabcentral/fileexchange/10093-fminspleas (accessed on 7 March 2022).
